# Supplementary material for: A clean and membrane-free chlor-alkali process with decoupled Cl2 and H2/NaOH production
Source: Nat Commun. 2018 Jan 30;9:438. doi: 10.1038/s41467-018-02877-x (PMC5789859; doi:10.1038/s41467-018-02877-x)
Supplement: Supplementary file 1 — Supplementary Information [file 41467_2018_2877_MOESM1_ESM.pdf]

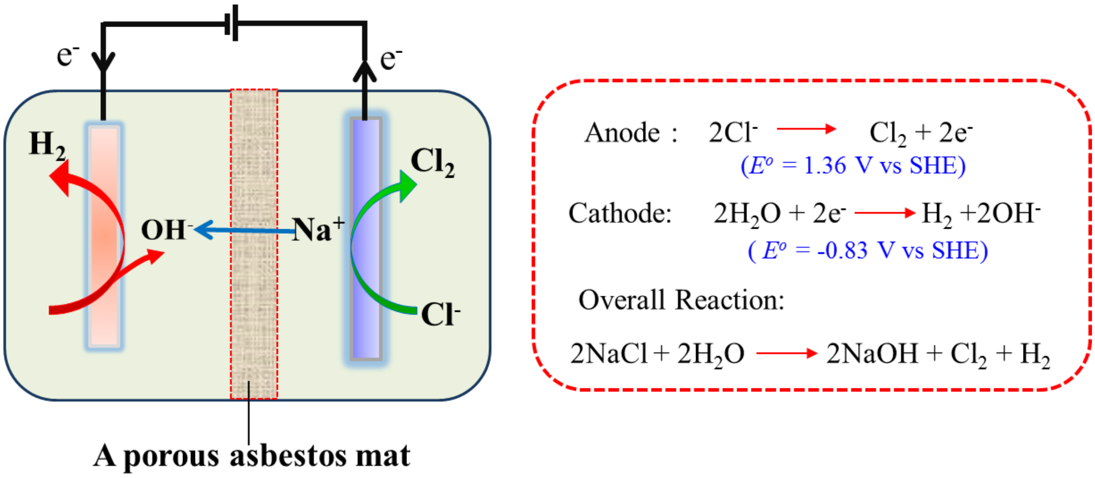

Supplementary Figure 1 | A schematic of the operation mechanism of the diaphragm cell. In the conventional diaphragm cell, a porous asbestos mat is used to separate the anodic reaction of CER and the cathodic reaction of HER, where NaOH is simultaneously formed in the cathodic compartment.[2]

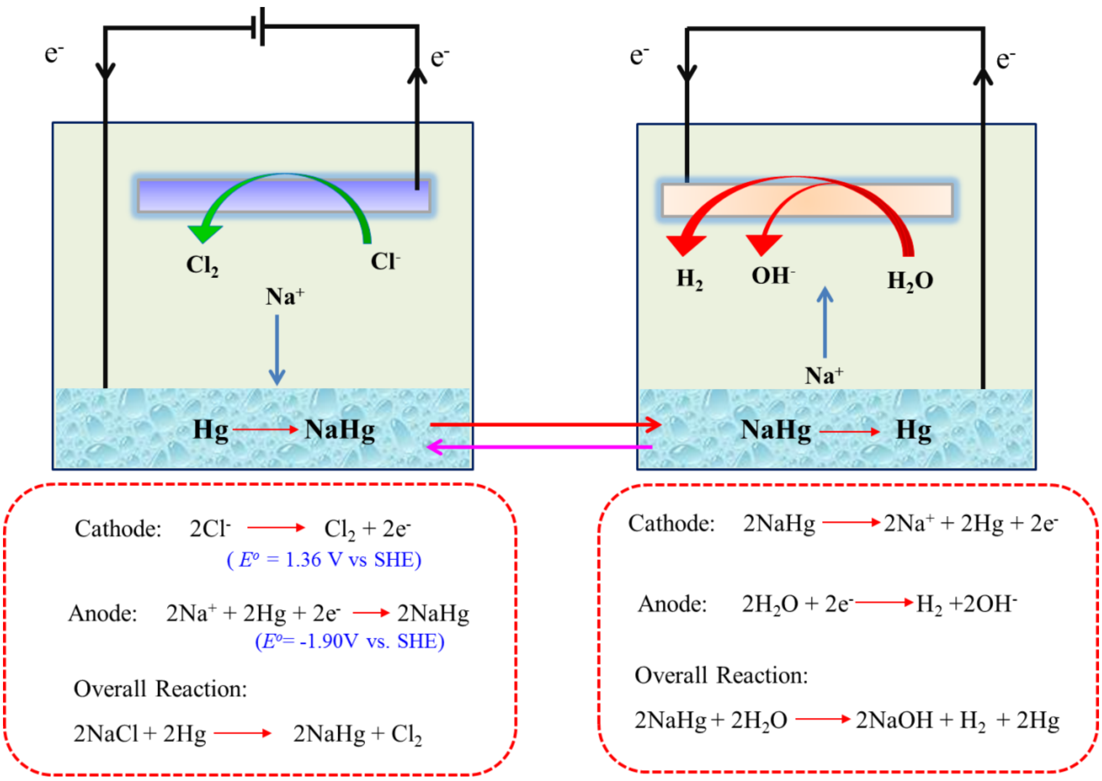

Supplementary Figure 2 | A schematic of the operation mechanism of the mercury cell. In the mercury cell, the CER reaction on the anode is coupled with the formation reaction of the sodium amalgam ($2Hg + 2Na^+ + 2e^- \rightarrow 2NaHg$) on the liquid mercury cathode. Then, the sodium amalgam is separated and used in the second cell to produce NaOH through the anodic reaction of the Na-ion release ($NaHg \rightarrow Na^+ + Hg + e^-$) and the cathodic reaction of HER reaction. As shown in supplementary Fig. 2, the theoretical driven voltage required for CER in mercury cell is as high as 3.26V [=1.36V - (-1.90) V].[2] In practical application, it is undoubted that the required driven voltage should be higher than this value (3.26V) because of polarization. Accordingly, it is well known that the energy consumption of mercury cell is higher than the diaphragm cell and the membrane cell.[3]

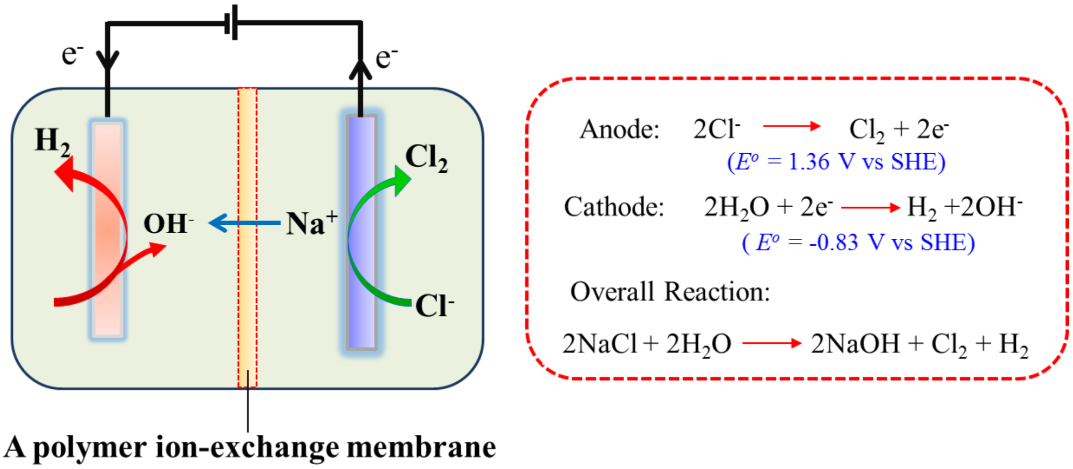

Supplementary Figure 3 | A schematic of the operation mechanism of the membrane cell. In present membrane cell, a polymer ion-exchange membrane (i.e. Nafion film) is used to separate the anodic reaction of CER and the cathodic reaction of HER, where NaOH is simultaneously formed in the cathodic compartment. [2]

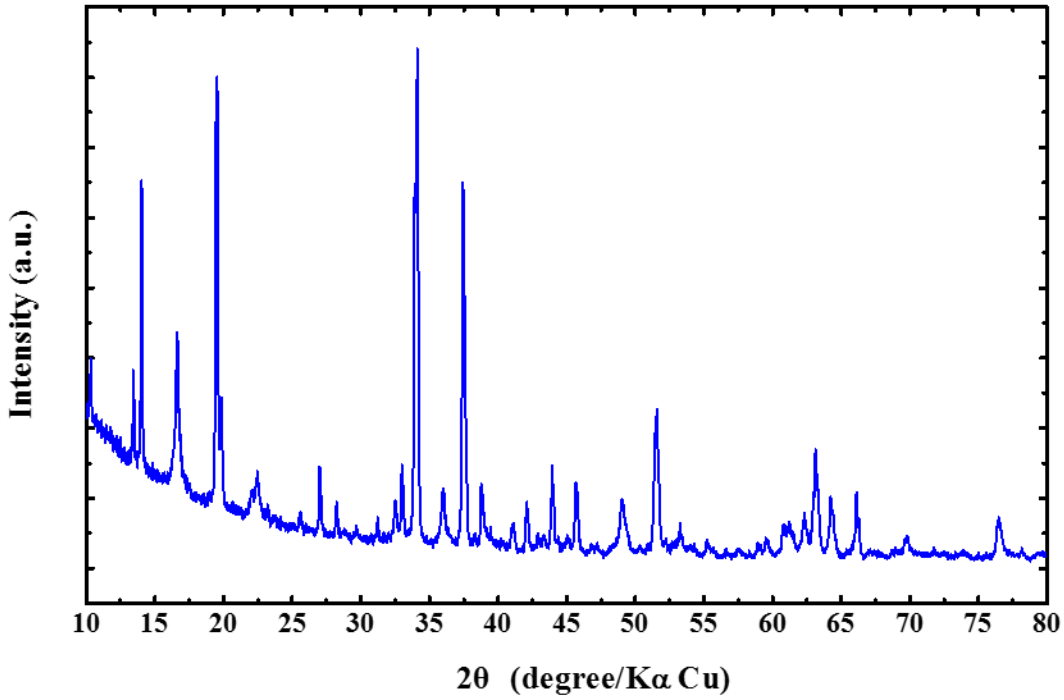

Supplementary Figure 4 | XRD pattern of the $Na_{0.44}MnO_2$ synthesized from the solid-state method. The patterns confirm the formation of well-crystallized $Na_{0.44}MnO_2$ (JCPDS No. 27-0750). All diffraction peaks can be indexed as an orthorhombic structure and *Pbam* space group, which is well consistent to the previously reported results.[4-8]

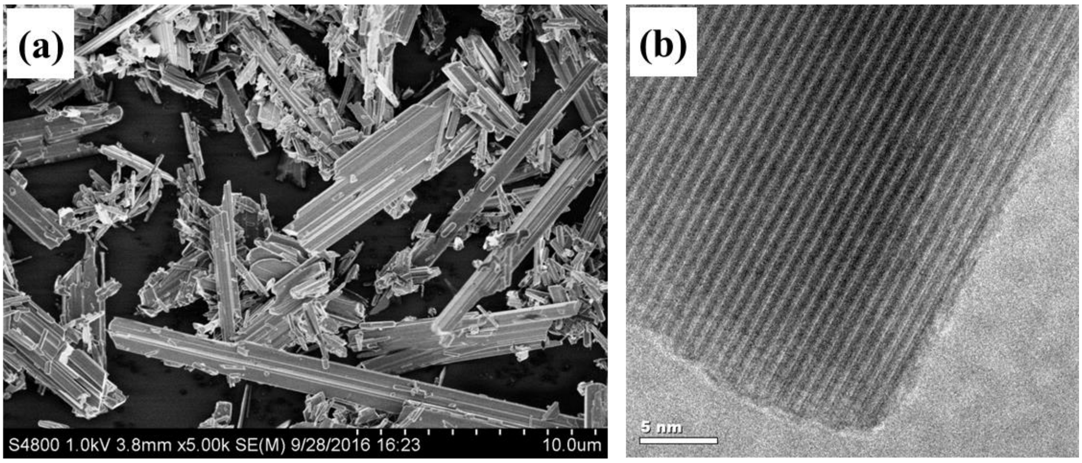

Supplementary Figure 5 | SEM (a) and HR-TEM (b) images of the $Na_{0.44}MnO_2$ powder. Most of the as-prepared $Na_{0.44}MnO_2$ particles are found to grow anisotropically into rod shapes and well crystallized. The HR-TEM image of the sample shows the ordered one-dimension lattice and suggests the single-crystalline structure.

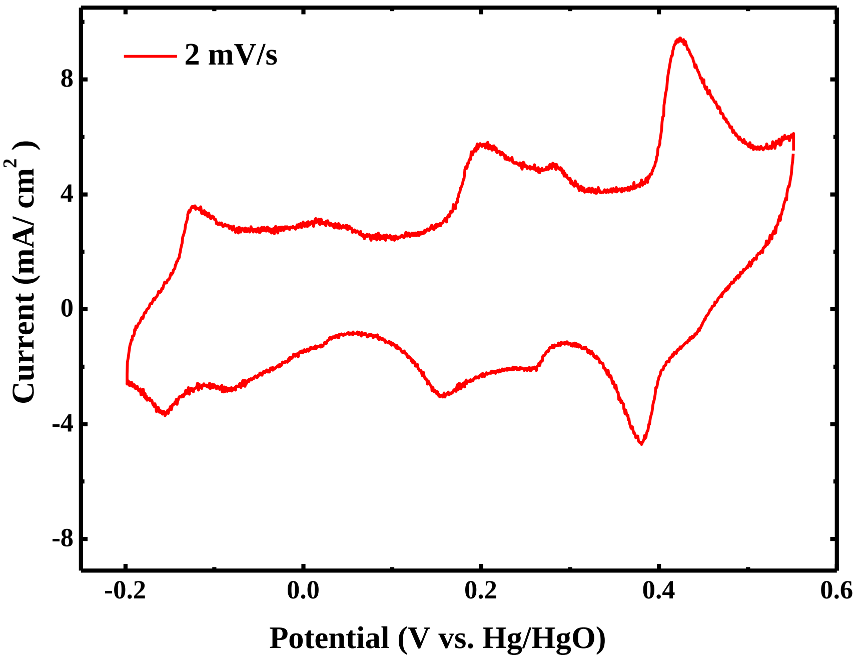

Supplementary Figure 6 | CV curve of the $Na_{0.44}MnO_2$ electrode in a 1M NaOH solution at a scanning rate of 2mV/s. The mass loading of $Na_{0.44}MnO_2$ is 7mg cm^{-2}.

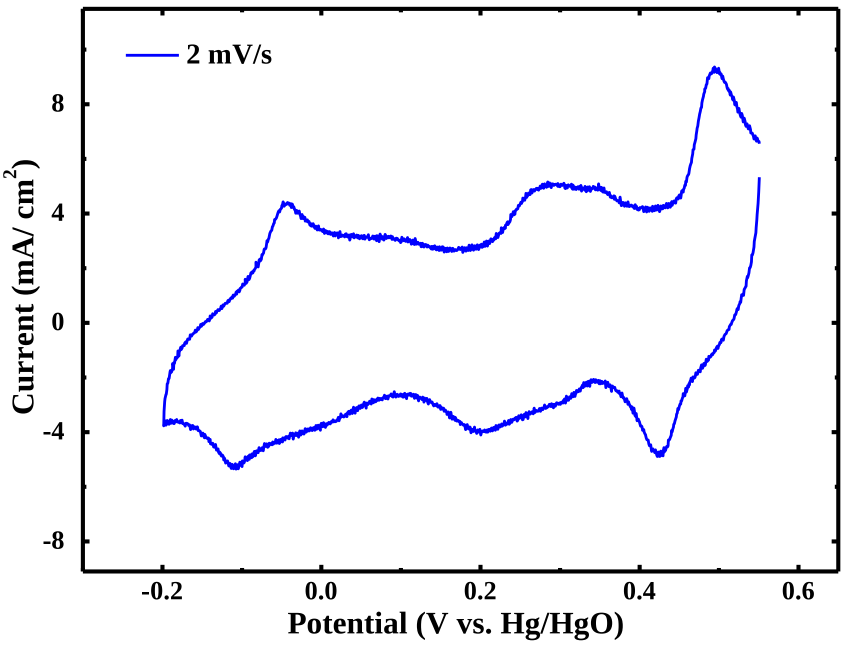

Supplementary Figure 7 | CV curve of the $Na_{0.44}MnO_2$ electrode in a saturated NaCl solution at a scanning rate of 2mV/s. The mass loading of $Na_{0.44}MnO_2$ is 7mg cm^{-2}.

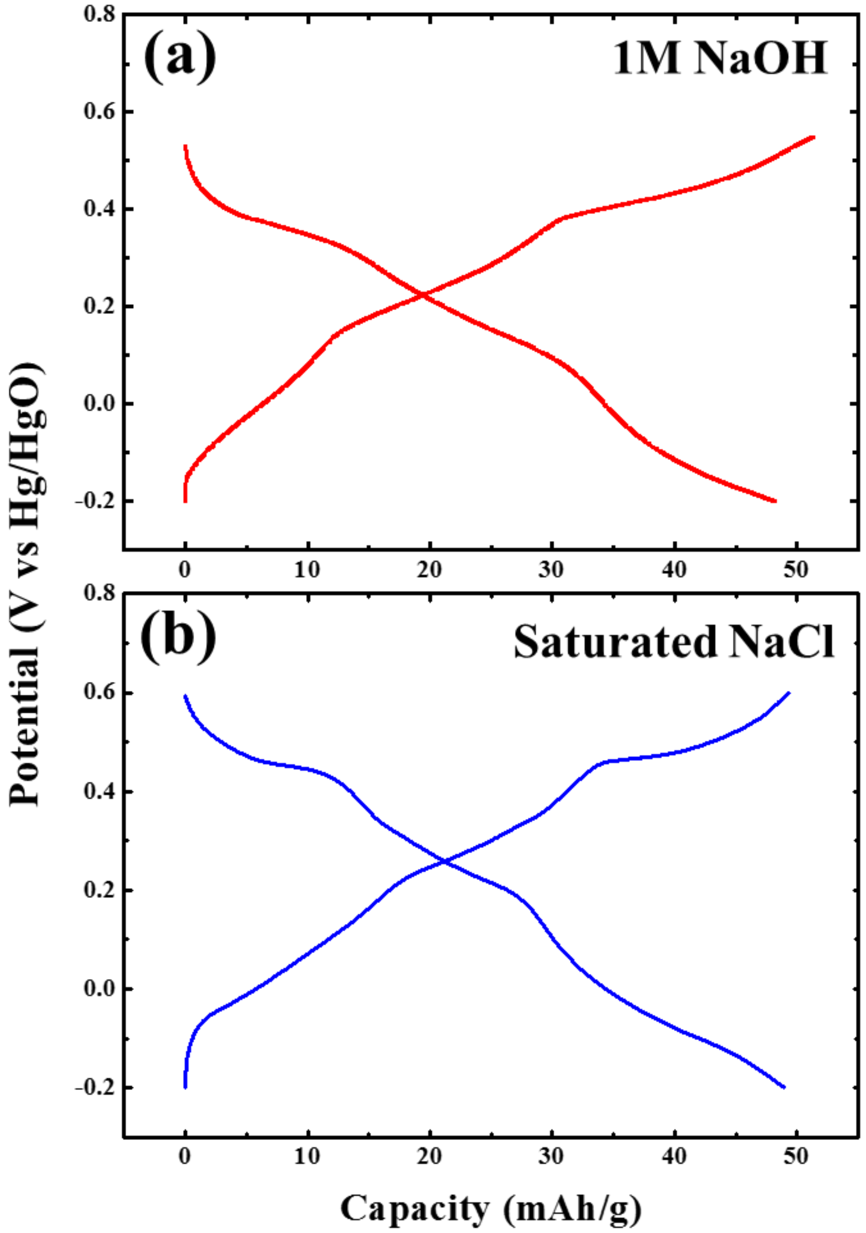

Supplementary Figure 8 | Galvanostatic charge/discharge curves of the $Na_{0.44}MnO_2$. (a) in a 1M NaOH solution and **(b)** in a saturated NaCl solution. Supplementary Fig. 8 exhibits the typical galvanostatic charge–discharge curves of the $Na_{0.44}MnO_2$ electrode at a current density of 0.1 A/g within a potential window of -0.2-0.55V (vs. Hg/HgO). A specific discharge capacity of about 48 mAh/g is obtained in both the 1M NaOH and the saturated NaCl solutions.

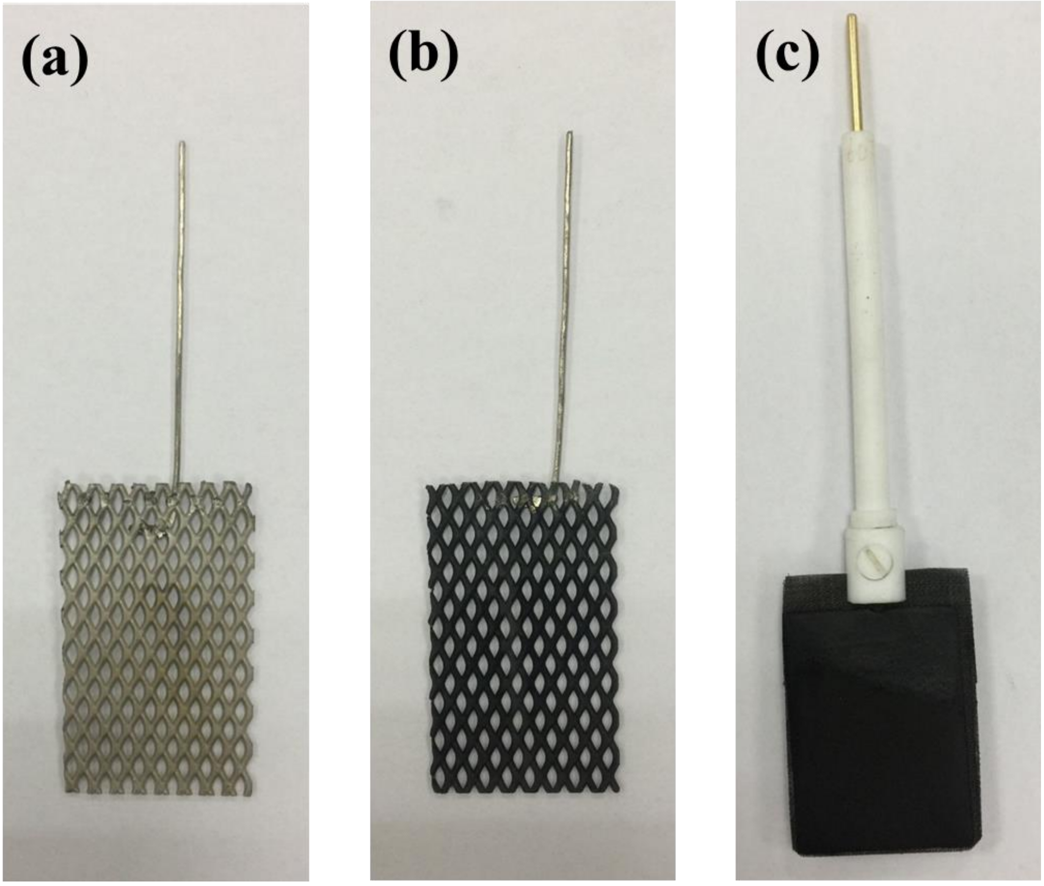

Supplementary Figure 9 | Photograph of the electrodes. (a) the commercial Pt coated Ti-mesh electrode, (b) the commercial RuO_2/IrO_2 coated Ti-mesh electrode and (c) the $Na_{0.44}MnO_2$ electrode. The size of all these electrodes is 2.5×4 cm^2.

Both the Pt and RuO_2/IrO_2 coated Ti-mesh electrodes are purchased from Baoji Zhiming Special Metal Co., LTD (China). [Tel. 86-0917-3122785; http://www.zmanode.com].

The $Na_{0.44}MnO_2$ electrode was prepared by mixing 70 wt % active materials, 8 wt % acetylene black, 8 wt % carbon nanotubes and 14 wt % polytetrafluoroethylene binder dispersed in isopropanol, which was then treated with a roll press machine to form a film. Finally, the film was pressed onto a titanium grid that served as a current collector. Mass loading of $Na_{0.44}MnO_2$ is ~200 mg cm^{-2}. The electrode was fixed by a platinum electrode holder during the two-step electrolytic process, for the convenience of transferring the electrode between the two different solutions.

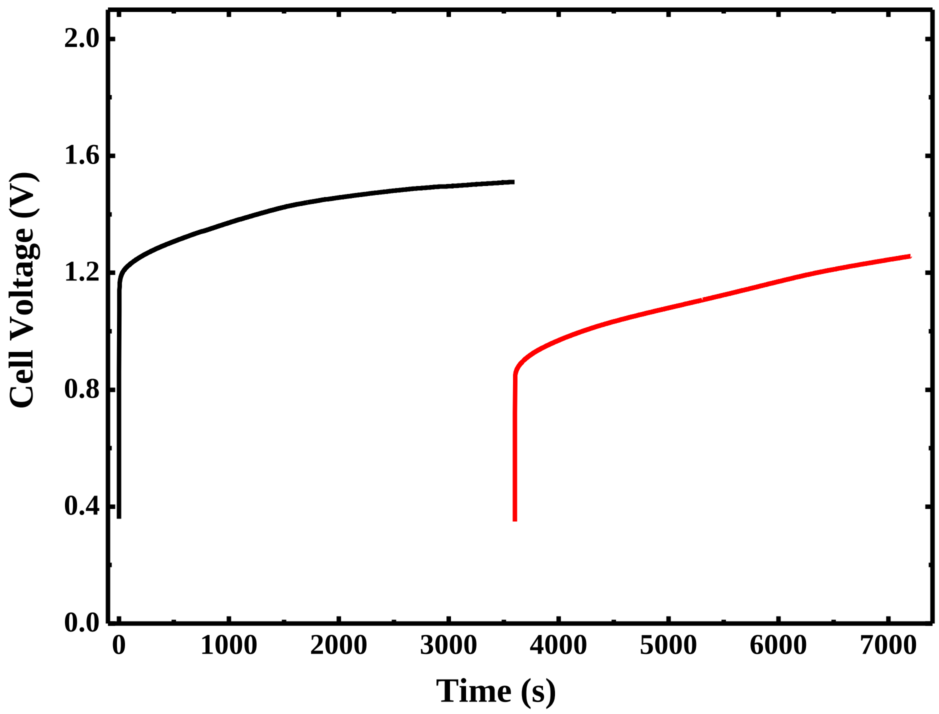

Supplementary Figure 10｜ Chronopotentiometry curves (cell voltage versus time) of the chlor-alkali processes at an applied current of 100mA with a step time of 3600s. The size of all these electrodes (Pt coated Ti mesh, RuO_2/IrO_2-coated Ti mesh and $Na_{0.44}MnO_2$ electrode) is 2.5×4 cm^2.

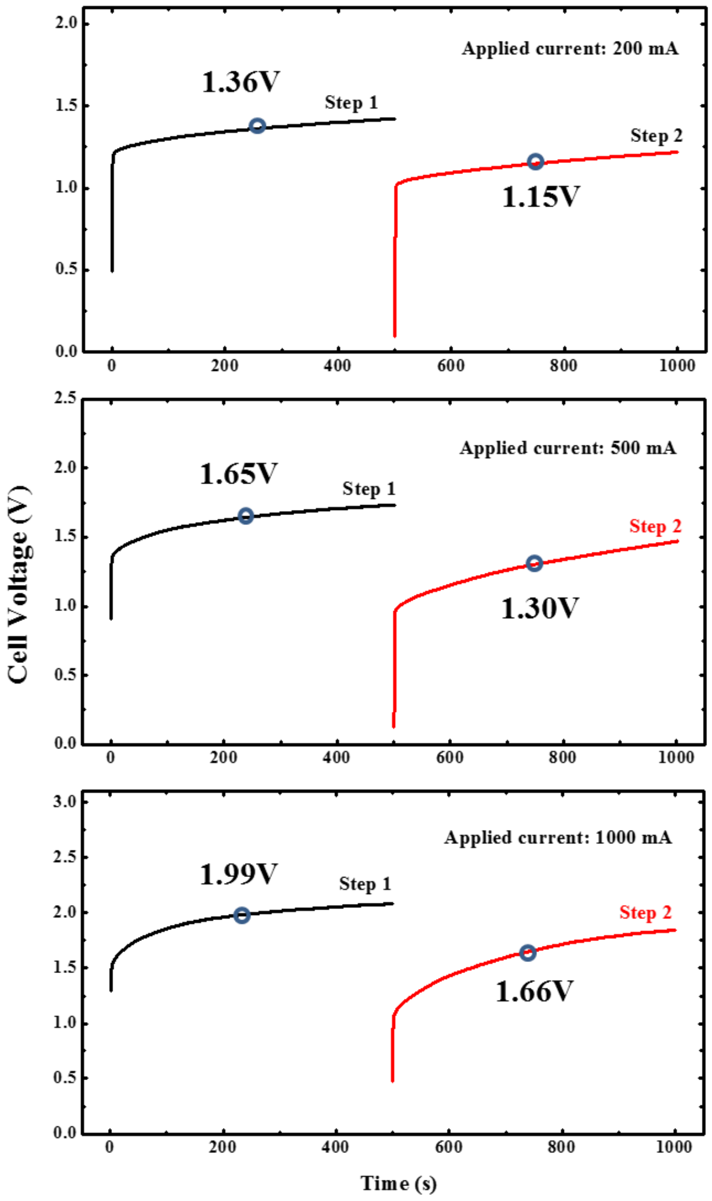

Supplementary Figure 11 | Chronopotentiometry curves (cell voltage versus time) of the chlor-alkali processes at higher currents of 200mA, 500mA and 1000mA. The size of all these electrodes (Pt coated Ti mesh, RuO_2/IrO_2-coated Ti mesh and $Na_{0.44}MnO_2$ electrode) is 2.5×4 cm^2.

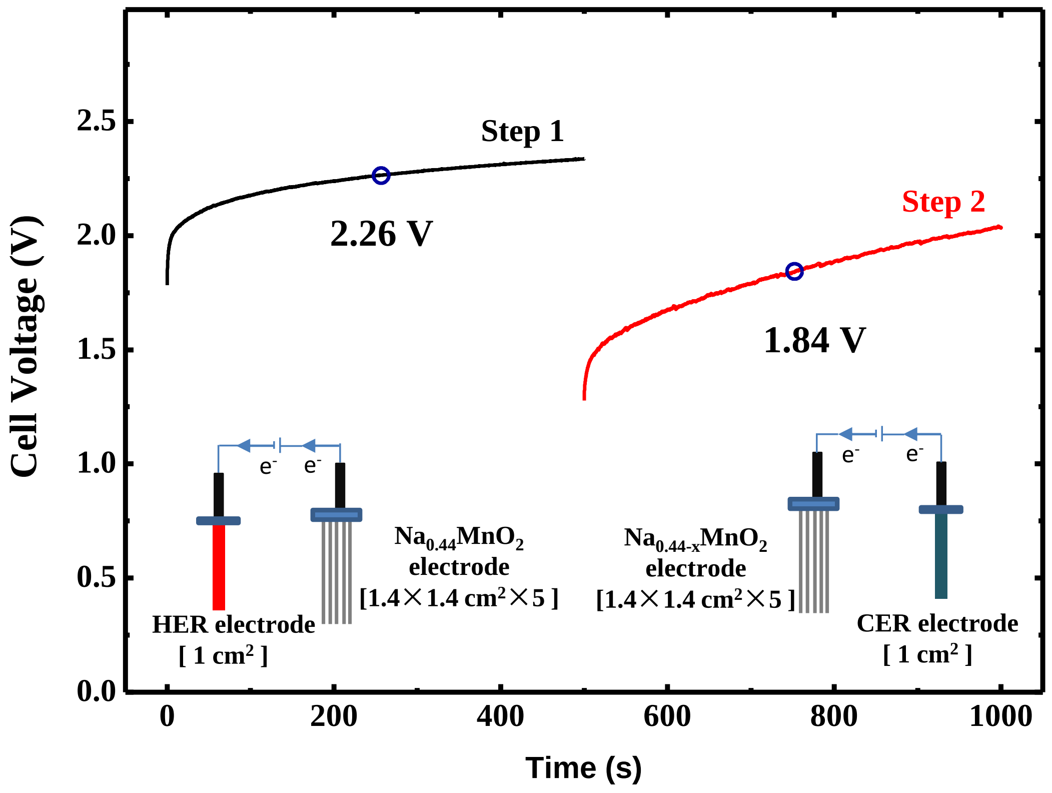

Supplementary Figure 12 | Chronopotentiometry curves of the chlor-alkali processes at an applied current of 500mA with a step time of 500s. [The HER electrode: 1×1 cm^2 Pt coated Ti mesh; The CER electrode: 1×1 cm^2 RuO_2/IrO_2-coated Ti mesh; The $Na_{0.44}MnO_2/Na_{0.44-x}MnO_2$ electrode (see inset): it is made up of 5 pieces of electrodes with total area of 10 cm^2 (=1.4 ×1.4 cm^2× 5)]. The applied current density on HER or CER is 500 mA cm^{-2}. Area ratio between the HER (or CER) electrode and the battery electrode is 1: 10.

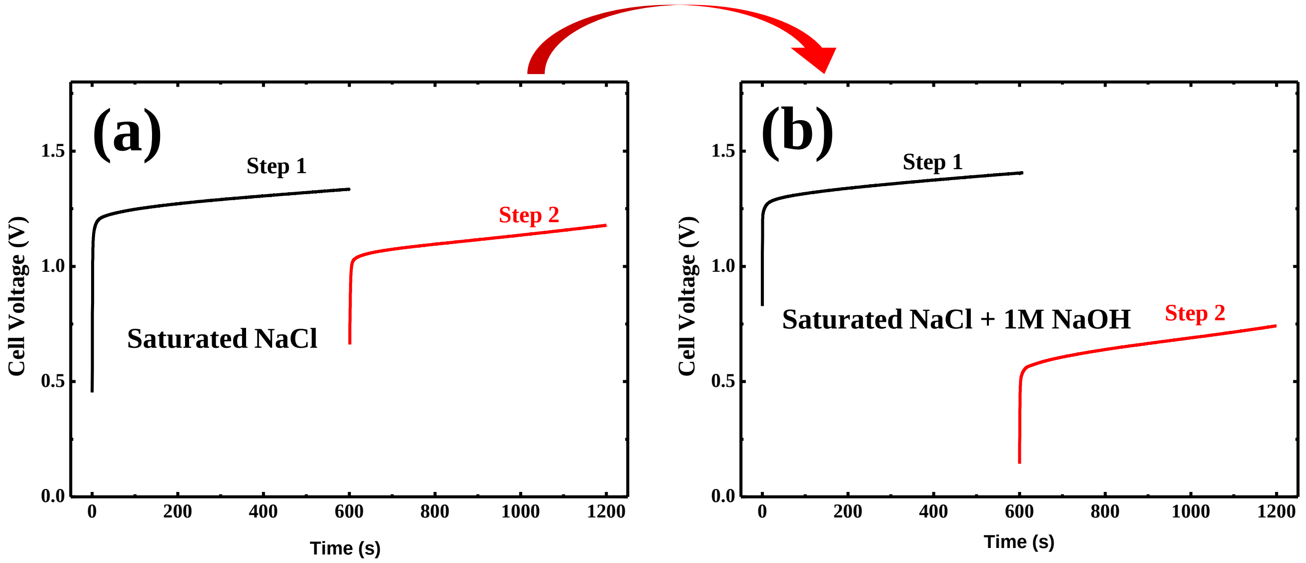

Supplementary Figure 13 | Chronopotentiometry curves of the chlor-alkali processes in one cell at an applied current of 100mA. (a) With a pure saturated NaCl solution as the electrolyte; (b) With a saturated NaCl and 1M NaOH solution as the electrolyte. The size of all these electrodes (Pt coated Ti mesh, RuO_2/IrO_2-coated Ti mesh and $Na_{0.44}MnO_2$ electrode) is 2.5×4 cm^2. In theory, the three electrodes in a single cell with a saturated NaCl electrolyte solution can be directly used to perform the Step 1 and Step 2, which is confirmed by supplementary Fig. 13a. However, the generated NaOH is dissolved in the brine electrolyte solution, indicating no pure NaOH stream is produced. Furthermore, with growth of cycle number of Steps 1 and 2, NaOH concentration in the electrolyte solution will increase step by step. Then, the undesired O_2 evolution reaction (OER) of $4OH^- \rightarrow O_2 + 2H_2O + 4e^-$ occurs on the CER electrode. For example, when there is 1 M NaOH in the electrolyte solution (Supplementary Fig. 13b), the achieved cell voltage of Step 2 is much lower than that in saturated NaCl solution (Supplementary Fig. 13a). This phenomenon is attributable to that the CER reaction ($2Cl^- \rightarrow Cl_2 + 2e^-$) has been totally replaced by OER reaction ($4OH^- \rightarrow O_2 + 2H_2O$) at lower potential. It is well known that in alkaline solution the potential of OER is lower than that of CER because of the high concentration of OH^-.

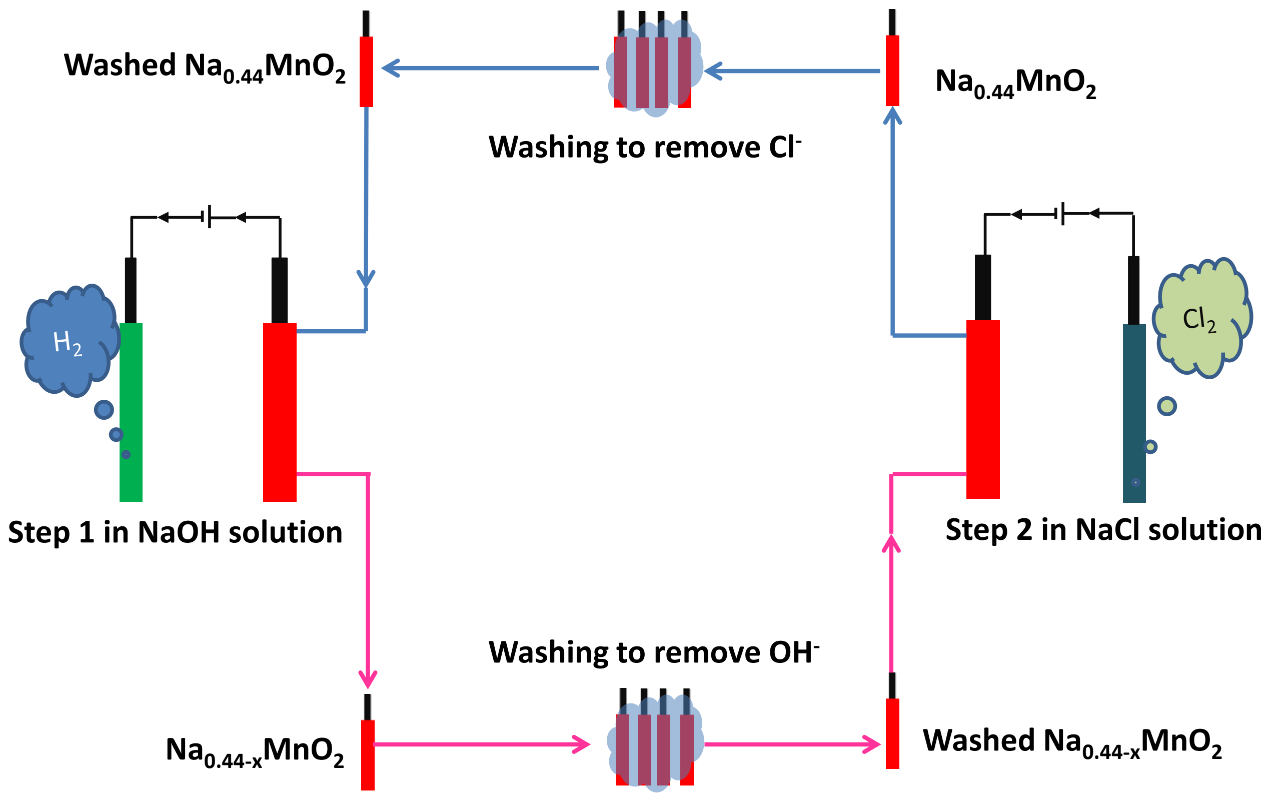

Supplementary Figure 14 | A conceptual process scheme of the two step chlor-alkali process.

As shown in supplementary Fig. 14, all the steps can be contentiously operated, in parallel with transferring of battery electrodes ($Na_{0.44}MnO_2/Na_{0.44-x}MnO_2$) alone with these arrows. The technology challenge facing the design and potential solution are summarized as following:

Firstly, the continuous transfer of battery electrodes needs advanced automation design. Herein, the electrode transfer technology in battery industry, such as mechanical rechargeable Zn-air battery, can be used as reference [9].

Secondly, washing process shown in supplementary Fig. 14 needs advanced purification technology. Generally, OH^- can be easily removed by washing with a diluent HCl solution. However, it is very difficult to totally remove Cl^- with washing process. As a result, some Cl^- may still present in the prepared NaOH solution. The similar issue exists in the diaphragm cell. Therefore, the purification technology in the old diaphragm cell is necessary for the decoupled process.

Finally, other technology issues must be based on the existing chlor-alkali processes that have been developed more than 100 years. It should be noted that both the hydrogen/chlorine evolution electrodes and corresponding reactions (HER in an alkaline solution/CER in a brine solution) of the decoupled process are as same as that of existing ones.

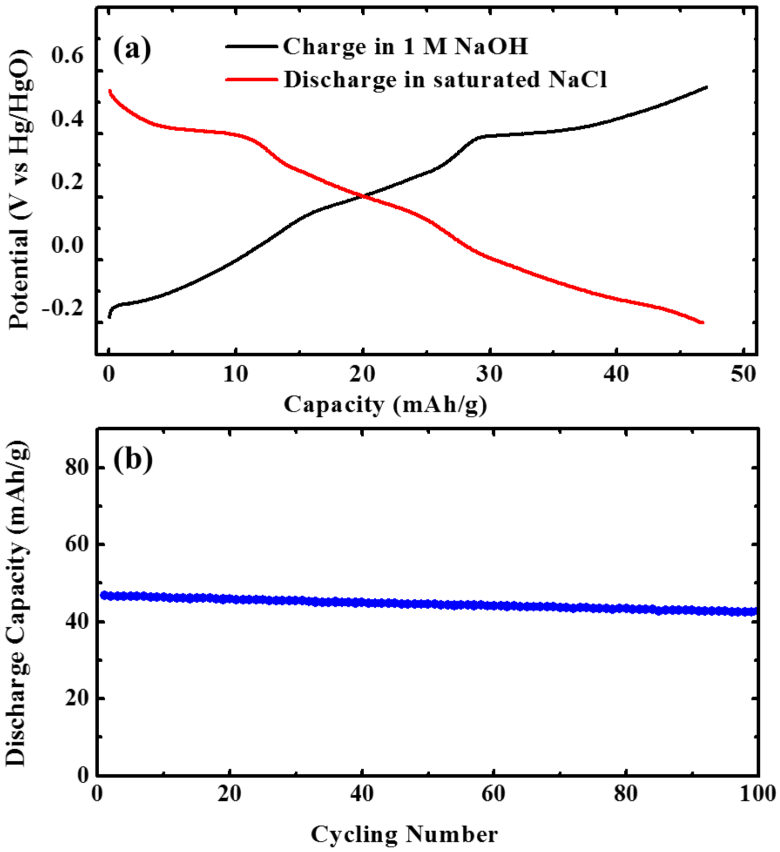

Supplementary Figure 15 | Cycle performance of the $Na_{0.44}MnO_2$ electrode tested at a current density of 100 mA g^{-1}. (a) Charge/discharge curves. (B) Cycle life (discharge capacity vs. cycle number). In this experiment, the charge and discharge were performed in the 1 M NaOH solution and the saturated NaCl solution, respectively. There is a washing step between the charge and discharge to remove the surface-adsorbed OH^- or Cl^-. As shown in Supplementary Fig. 15a, the electrode exhibits a reversible charge/discharge capacity of ~47 mAh/g. In addition, nearly 91% of the initial capacity was retained after 100 cycles (Supplementary Fig. 15b). The mass loading of $Na_{0.44}MnO_2$ is ~7mg cm^{-2}.

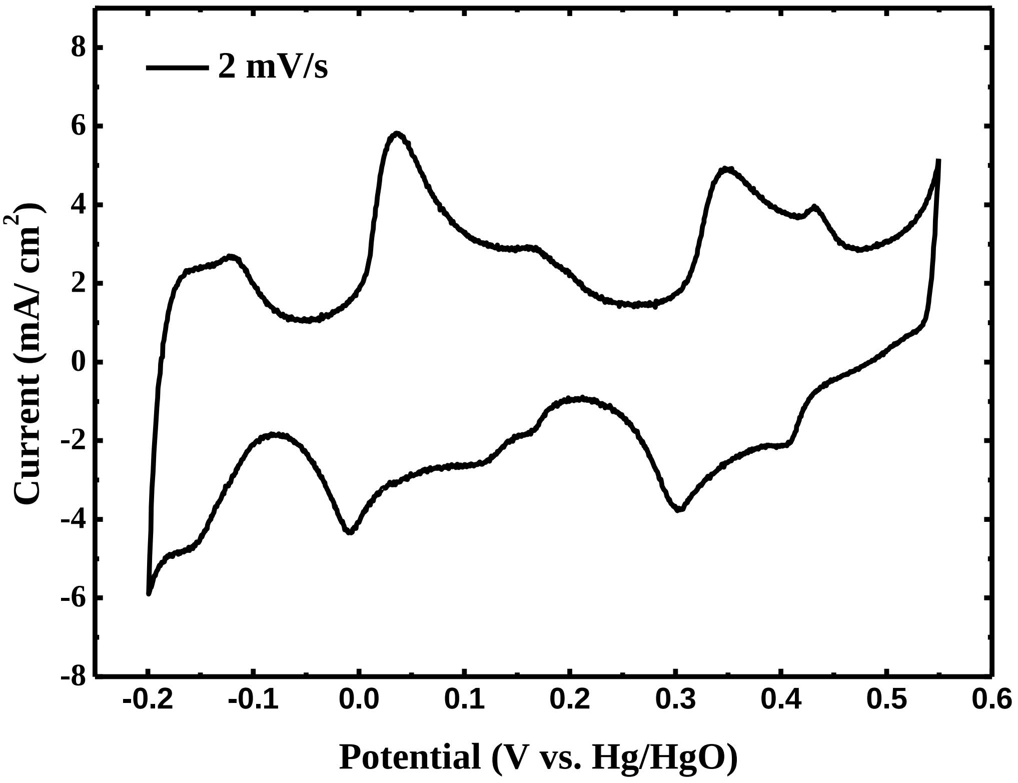

Supplementary Figure 16 | CV curve of the $Na_{0.44}MnO_2$ electrode in a 10M NaOH solution at a scanning rate of 2mV/s. The mass loading $Na_{0.44}MnO_2$ is 7mg cm^{-2}. As shown supplementary Fig. 16, the achieved electrochemical profile is as same as that in 1M NaOH.

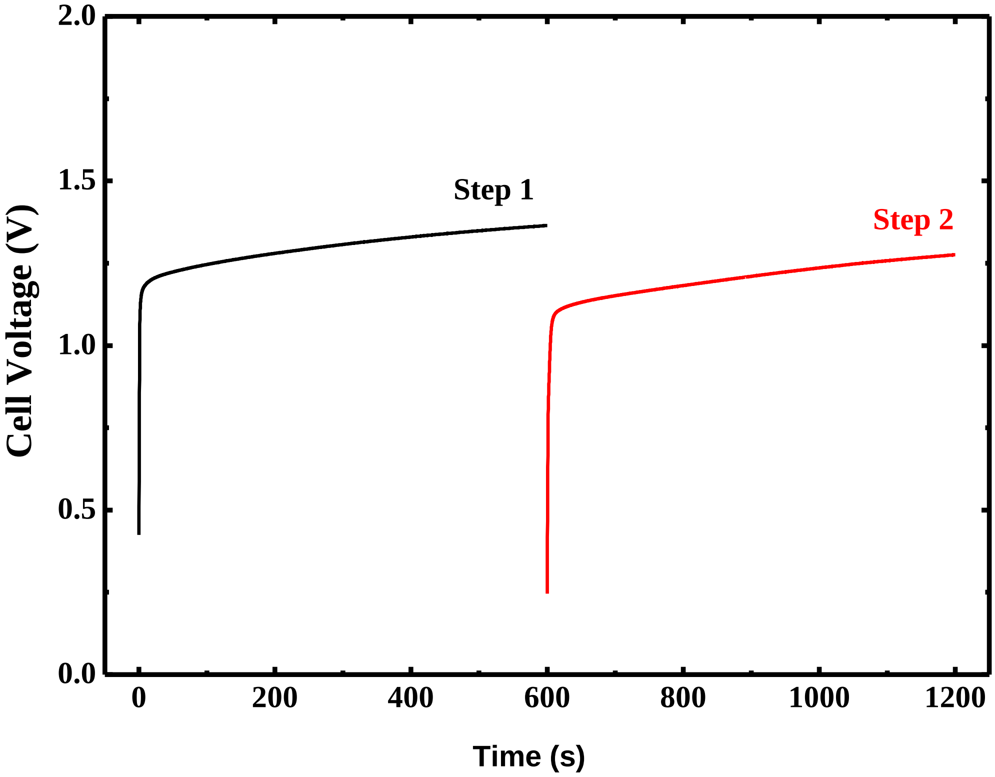

Supplementary Figure 17 | Chronopotentiometry curves (cell voltage versus time) of the chlor-alkali processes, where the 10M (~30 wt. %) NaOH electrolyte solution and the saturated NaCl electrolyte were employed to performance Step 1 and Step 2, respectively. The size of all these electrodes (Pt coated Ti mesh, RuO_2/IrO_2-coated Ti mesh and $Na_{0.44}MnO_2$ electrode) is 2.5×4 cm^2.The achieved results in supplementary **Fig. 17** (i.e. electrochemical profile of both steps) are as same as that performed in the 1M NaOH and the saturated NaCl solutions.

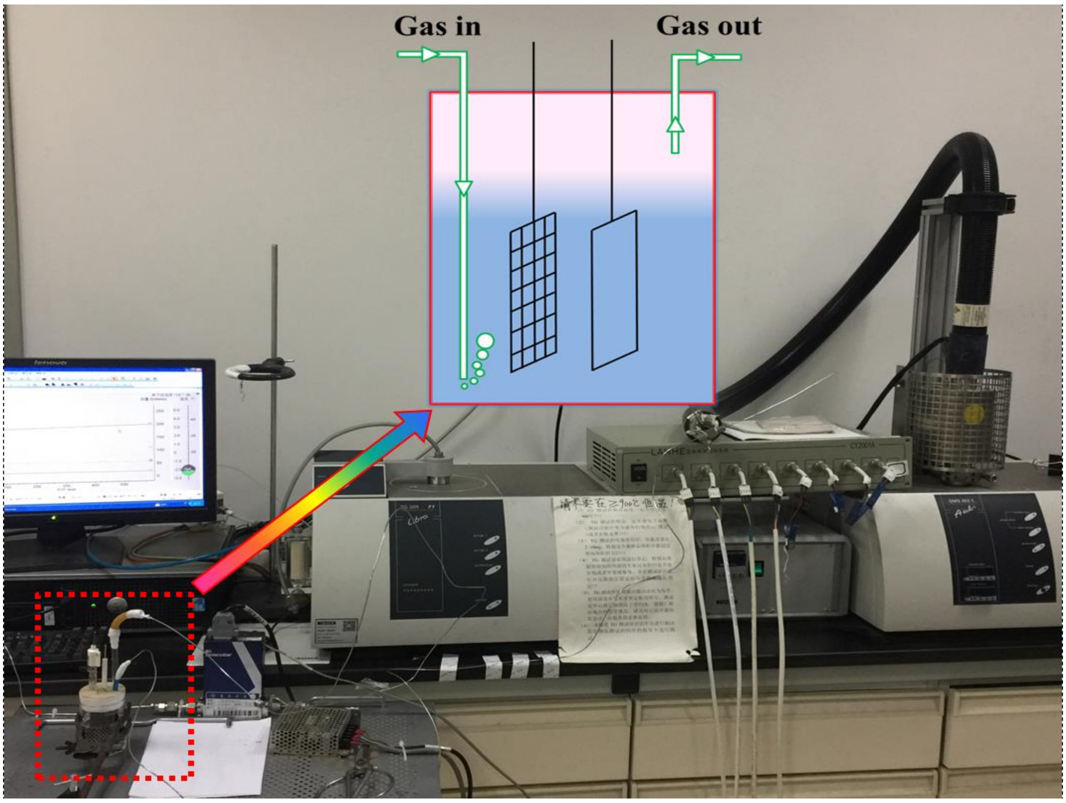

Supplementary Figure 18 | Schematic illustration and photo of the electrolysis cell connected to the mass spectrometer devices.

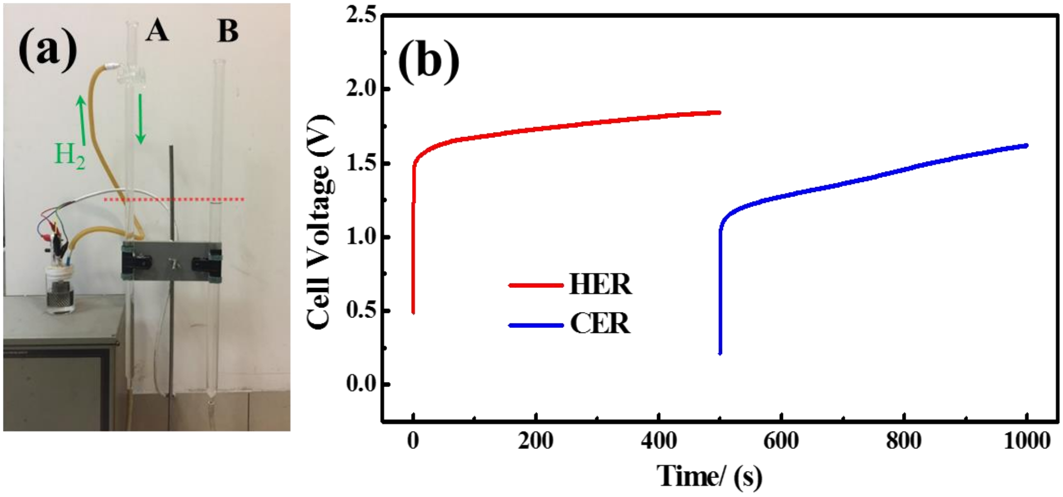

Supplementary Figure 19 | Gas volume measurement. (a) The photo of the drainage device for gas volume measurement and (b) the corresponding electrochemical profile during gas volume measurement. As shown, the generated gas can be collected in tube-A. Tube-B should be shifted during the measurement to keep the liquid level as same as that in tube-A, then the volume of the generated gas can be read from the scale on tube-A. The saturated NaCl solution in the tube-A and B was used to seal the gas.

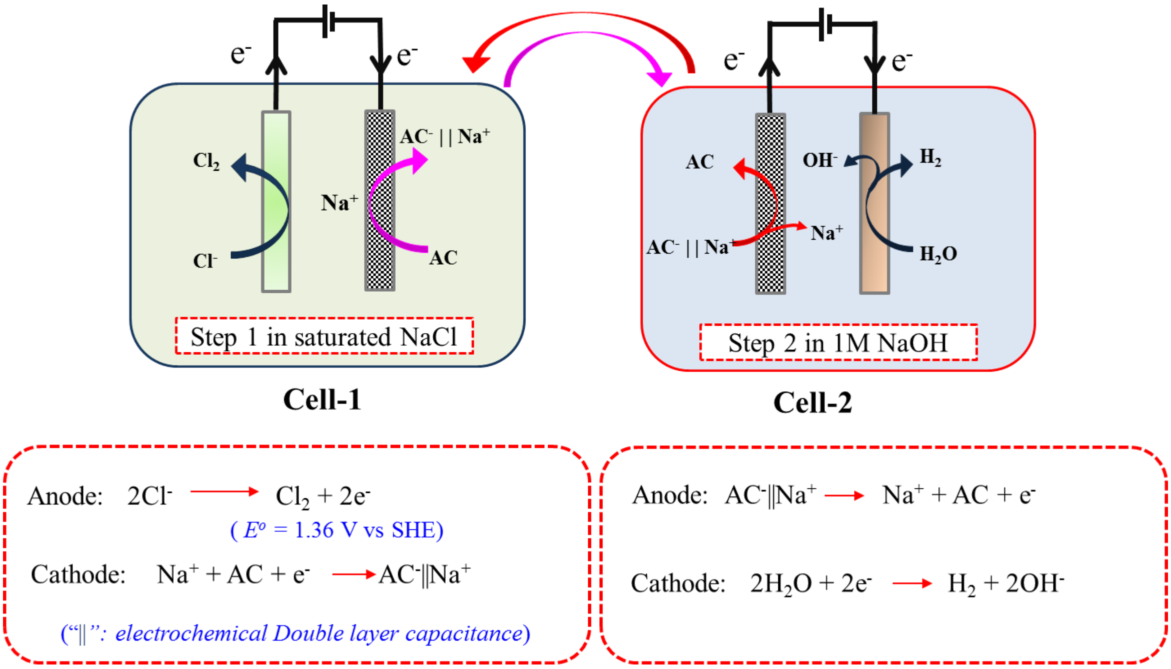

Supplementary Figure 20 | Operation mechanism of two-step chlor-alkali electrolysis using Na^+ electrochemical adsorption/desorption (= electrochemical double layered capacitance) on activated carbon (AC) as a charge mediator. In Step 1, the CER reaction on the anode (RuO_2/IrO_2 coated Ti mesh) is coupled to the electrochemical adsorption of Na^+ on the AC based cathode ($Na^+ + AC + e^- \rightarrow AC^-||Na^+$; "||" refers to *double layer*) in Cell-1 containing the saturated NaCl solution. Then, the AC electrode is separated and used for Step 2 in Cell-2 containing 1M NaOH, which involves the electrochemical desorption of Na^+ on the AC based anode($AC^-||Na^+ \rightarrow Na^+ + AC + e^-$) and the HER on the cathode (Pt coated Ti mesh).

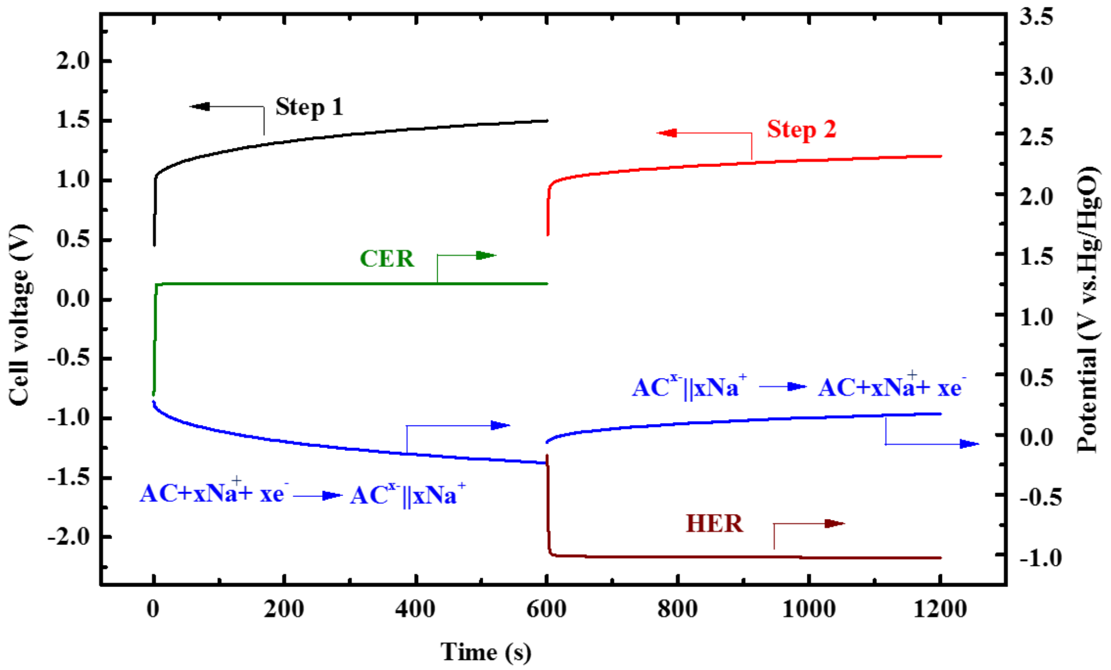

Supplementary Figure 21 | Chronopotentiometry curves (cell voltage vs. time) of the electrolysis cell using AC electrode as a charge mediator. The commercial Pt coated Ti-mesh electrode (2.5×4 cm^2), commercial RuO_2/IrO_2 coated Ti-mesh electrode (2.5×4 cm^2) and AC electrode (2.5×4 cm^2; mass loading of AC is 200mg cm^{-2}) were used to build the electrolysis cells according to **Extended Data Fig. 21**. The AC electrode was prepared by mixing 80 wt % AC, 10wt % acetylene black and 10 wt% polytetrafluoroethylene binder dispersed (PTFE) in isopropanol, which was then treated with a roll press machine to form a film. Finally, the film was pressed onto a titanium grid that served as a current collector. supplementary Fig. 21 gives the Chronopotentiometry curve of the electrolysis cells at a constant applied current of 100 mA with two steps: Step 1 is Cl_2 production process (the black line); Step 2 is H_2 production process (the red line). Chronopotentiometry data ((potential vs. time) of the CER electrode (the green line), the AC electrode (the blue line) and the HER electrode (the wine line) are also given in supplementary Fig. 21. As shown in supplementary Fig. 21, step 1 exhibits an average cell voltage of about 1.38 V, which arises from the average potentials difference between the anodic oxidation of Cl^- ($Cl^- \rightarrow Cl_2$) and the cathodic absorption of Na^+ on the AC electrode. In consequent step 2 (i.e. H_2 + NaOH production process), the average cell voltage is 1.14V, which is equal to the average potential difference between the anodic potential 0.12V (vs. Hg/ HgO) of Na^+ desorption from the AC electrode and the cathodic potential -1.02V (vs. Hg/HgO) of H_2O reduction ($H_2O \rightarrow H_2$)

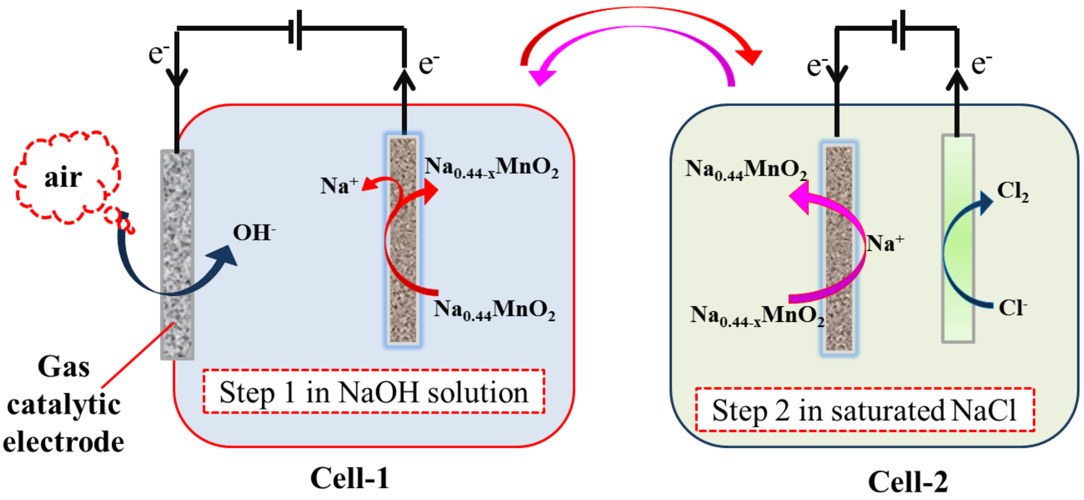

Supplementary Figure 22 | A schematic illustration of the decoupled operation mechanism of the electrolysis cell with an oxygen depolarized cathode. Step 1 involves the cathodic reduction of O_2 ($O_2 + 2H_2O + 4e^- \rightarrow 4OH^-$) on gas catalytic electrode and the anodic Na^+ de-intercalation from $Na_{0.44}MnO_2$ electrode ($Na_{0.44}MnO_2 \rightarrow Na_{0.44-x}MnO_2 + xNa^+ + xe^-$) in a NaOH solution (Cell-1), and Step 2 depends on the cathodic Na^+ intercalation in $Na_{0.44-x}MnO_2$ electrode ($Na_{0.44-x}MnO_2 + xNa^+ + xe^- \rightarrow Na_{0.44}MnO_2$) and the anodic Cl_2 evolution reaction on CER electrode ($2Cl^- \rightarrow Cl_2 + 2e^-$) in a saturated NaCl solution (Cell-2). The Step 1 and Step 2 can be cycled by moving $Na_{0.44}MnO_2$/$Na_{0.44-x}MnO_2$ electrode between cell-1 for Step 1 and cell-2 for Step 2.

Supplementary Table 1 Experimental data of H_2 evolution and corresponding Faradaic efficiencies obtained from 5 repeated experiments.

	V_{gas} (mL) **	$V_{dissolved}$ (mL)*	V_{total} (mL)***	E_{H2}****
#1	11.4	1.1	12.5	99.2%
#2	11.7	1.1	12.8	101.6%
#3	11.6	1.1	12.7	100.8%
#4	11.7	1.1	12.8	101.6%
#5	11.6	1.1	12.7	100.8%

*: The dissolution of H_2 in NaOH electrolyte solution is calculated according to the SOLUBILITY DATA SERIES HYDROGEN AND DEUTERIUM [10]. The calculation is given as $K_{cs\alpha}/dm^3\ mol^{-1} = (1/(c/mol\ dm^{-3}))\ log\ (\alpha^o/\alpha)$, where $K_{cs\alpha}$ is salt effect parameter; c is the electrolyte concentration in mol dm^{-3}; α^o and α are the Bunsen coefficients in pure water and electrolyte solution, respectively. $K_{cs\alpha}$ and α^o at 298.15K and 1 atm can be obtained from IUPAC solubility data series [10] [$K_{cs\alpha}$ = 0.137 for 1M NaOH (aq); α^o =1.77×10^{-2} ($H_{2\ volume\ at\ standard\ condition}$/1M $NaOH_{volume\ at\ 298.15K\ and\ 1\ atm}$; *Bunsen coefficient: the volume of saturating gas, V_1, reduced to T° = 273.15 K, p°=1 atm, which is absorbed by unit volume V_2* of pure solvent at the temperature of measurement* [11]]. The experiment temperature is 296.65 K that is very close to 298.15K. Based on above equation, the calculated α is 1.29×10^{-2}. Accordingly, 1.03 mL (=$1.29\times10^{-2}\times80$ mL) H_2 under standard condition (T° = 273.15 K, p°=1 atm) can be dissolved in 80 mL of 1M NaOH at experiment temperature. Assuming the gas is ideal, we can calculate that ~1.1 mL H_2 at real condition (296.65K, 1atm) can be dissolved in 80 mL 1M NaOH solution at 296.65K, 1atm.

**: The V_{gas} (mL) value is obtained by a drainage method (experiment operator: Dr. Mengyan Hou).

***: $V_{total} = V_{gas} + V_{dissolved}$.

****: Faradaic Efficiency for H_2 production (E_{H2}) is calculated by (V_{total}/V_{theory}). V_{theory} (12.6 mL) is calculated by ($I\times t = n\times z\times F$ *and* $P\times V = n\times R\times T$; I is 200 mA, t is 500s, P is 1 atm, T is 296.65 K).

As shown in Supplementary Table 1, the average Faradaic efficiency for H_2-production in decouple process is 100.8 %. The average error (*d*) is 0.6 % [=1/5 × (|99.2%-100.8%| + |101.6%-100.8%| + |100.8%-100.8%| + |101.6 %-100.8%| + |100.8%-100.8% |)].

Supplementary Table 2 Experimental data of Cl_2 evolution and corresponding Faradaic efficiencies obtained from 5 repeated experiments.

	V_{gas} (mL)*	$V_{dissolved}$ (mL)**	V_{total} (mL)***	E_{Cl2}****
#1	4.5	6.6	11.1	88.1%
#2	4.8	6.7	11.5	91.3%
#3	4.6	6.8	11.4	90.5%
#4	4.6	6.5	11.1	88.1%
#5	4.4	7.3	11.7	92.9%

*: The V_{gas} (mL) value is obtained by a drainage method (experiment operator: Dr. Mengyan Hou).

**: The dissolved Cl_2 (n, mol) that undergoes immediate hydrolysis is obtained by the titration method. The value of n is converted into the volume of dissolved Cl_2 through $P \times V = n \times R \times T$, where P is 1 atm, and T is the experiment temperature of 296.65 K.

***: $V_{total} = V_{gas} + V_{dissolved}$.

****: Faradaic Efficiency for Cl_2-production (E_{Cl2}) is calculated by (V_{total}/V_{theory}). V_{theory} (12.6 mL) is calculated by ($I \times t = n \times z \times F$ and $P \times V = n \times R \times T$; I is 200 mA, t is 500s, P is 1 atm, T is 296.65 K).

It can be detected from Supplementary Table 2 that the average Faradaic efficiency for Cl_2 production in the decouple process is 90.2 %. The average error (d) is 1.7 %=1/5×(|88.1%-90.2%| + |91.3%-90.2%|+ |90.5%-90.2%%| + |88.1 %-90.2% %| + |92.9%-90.2 %%|)]. The lower Faradaic efficiency should be attributed to the side reaction of Cl_2 reduction at the surface of $Na_{0.44}MnO_2$ electrode. This point is further confirmed by the membrane cell.

Supplementary Table 3 Experimental data of H_2/Cl_2 evolution from the membrane cell and corresponding Faradaic efficiencies obtained from 5 repeated experiments.

*H_2**	V_{gas} (mL)	$V_{dissolved}$ (mL)	V_{total} (mL)	E_{H2}
#1	11.7	1.1	12.8	101.7%
#2	11.4	1.1	12.5	99.3%
#3	11.5	1.1	12.6	100.1%
#4	11.6	1.1	12.7	100.9%
#5	11.6	1.1	12.7	100.9%

*Cl_2***	V_{gas} (mL)	$V_{dissolved}$ (mL)	V_{total} (mL)	E_{H2}
#1	5.1	7.1	12.2	96.8%
#2	5.2	7.1	12.3	97.6%
#3	5.0	7.2	12.2	96.8%
#4	5.1	7.3	12.4	98.4%
#5	5.1	7.2	12.3	97.6%

*: Volume of H_2 is measured by the same way that given in Supplementary Table 1.

**: Volume of Cl_2 is measured by the same way that given in Supplementary Table 2.

Supplementary Methods

In situ differential electrochemical mass spectrometry: A quadrupole mass spectrometer (NETZSCH QMS 403 C) with a leak inlet was applied to measure the gas evolution of the total chlor-alkali electrolysis process at a constant applied current of 200 mA. The electrolysis process was performed using a Solarton Instrument Model 1287 electrochemical interface. As shown in **Supplementary Figure 18**, the mass spectrometer was connected to the new-type alkaline water electrolysis cell with two tubes as the purge/carrier gas inlet and outlet. A pure Ar gas stream was used as the purge gas before electrolysis and the carrier gas during the electrolysis process. The gas flows were typically 10 mL min^{-1}. Before the online gas analysis, the system was purged with a pure Ar stream for 1.5 h. The system was purged with a pure Ar stream for 1 h before Step 1 began. The duration time of Step 1 was 30 min. After the end of Step 1, a rest step of 130 min was used with a pure Ar stream to eliminate the remnant H_2 in the system. Then, the resulting $Na_{0.44-x}MnO_2$ electrode that was formed in Step 1 was washed with a NaCl solution to remove the surface-adsorbed NaOH. The washed $Na_{0.44-x}MnO_2$ electrode was moved to another cell for Cl_2 production (Step 2) and corresponding DEMS analysis.

Iodometric Titration: the total chlorine evolved during the CER process was obtained by summing the gaseous chlorine from the drainage method and the amount of solved chlorine in the NaCl solution. The total available chlorine in the solution (including free and combined available chlorine) was determined by an iodometric titration using potassium iodide (100 g/L), soluble starch (5 g/L), sulphuric acid (2 mol/L) and sodium thiosulfate (0.025 mol/L) [1]. The details of the titration are as follows: (1) Pipette 20 mL of the electrolysed sample solution into a 250-mL conical flask and add approximately 20 mL H_2SO_4 and 20 mL KI. After several minutes, I^- in this solution is oxidized into I_3^-, and the solution exhibits a yellow colour. (2) Add $Na_2S_2O_3$ (standardized before use) from a buret until the yellow colour of the liberated iodine is almost discharged. (3) Add 1 mL starch solution and titrate until the blue colour disappears. [The result was corrected by determining the blank contribution from reagent impurities and repeating with further aliquots of the sample solution.]

Supplementary References

1 Clescerl, L. S. *Standard Methods for the Examination of Water and Wastewater,* 20th Ed. American Public Health Association, (1998).

2 O'Brien, T. F., Bommaraju, T. V. & Hine, F. Handbook of Chlor-Alkali Technology. (2005).

3 Industrial Electrochemistry and Electrochemical Engineering Division H. H. Dow Memorial Student Achievement Award. *Electrochem. Soc. Interface* **24**, 72-72 (2015).

4 Kim, S.-W., Seo, D.-H., Ma, X., Ceder, G. & Kang, K. Electrode Materials for Rechargeable Sodium-Ion Batteries: Potential Alternatives to Current Lithium-Ion Batteries. *Adv. Energy Mater.* **2**, 710-721 (2012).

5 Wang, Y. *et al.* Ti-substituted tunnel-type $Na_{0.44}MnO_2$ oxide as a negative electrode for aqueous sodium-ion batteries. *Nat. Commun.* **6**, 6401 (2015).

6 Cao, Y. *et al.* Reversible sodium ion insertion in single crystalline manganese oxide nanowires with long cycle life. *Adv. Mater.* **23**, 3155-3160 (2011).

7 Sauvage, F., Laffont, L., Tarascon, J. M. & Baudrin, E. Study of the insertion/deinsertion mechanism of sodium into $Na_{0.44}MnO_2$. *Inorg. Chem.* **46**, 3289-3294 (2007).

8 Whitacre, J. F., Tevar, A. & Sharma, S. $Na_4Mn_9O_{18}$ as a positive electrode material for an aqueous electrolyte sodium-ion energy storage device. *Electrochem. Commun.* **12**, 463-466 (2010).

9 Caramia, V. & Bozzini, B, Materials science aspects of zinc–air batteries: a review, *Mater Renew Sustain Energy* **3**, 28, (2014).

10 C. L. Young, Ed., IUPAC Solubility Data Series, Vol. 5/6, *Hydrogen and Deuterium*, Pergamon Press, Oxford, England, 1981

11 Eike, B., Matthew, M., Friedrichs, G. & Julie, L., The Bunsen gas solubility coefficient of ethylene as a function of temperature and salinity and its importance for nitrogen fixation assays, *Limnol. Oceanogr-Meth.*, **2**, 282-288 (2004).
