# Supplementary material for: A clean and membrane-free chlor-alkali process with decoupled Cl2 and H2/NaOH production
Source: Nat Commun. 2018 Jan 30;9:438. doi: 10.1038/s41467-018-02877-x (PMC5789859; doi:10.1038/s41467-018-02877-x)
Supplement: Supplementary file 3 — Description of Additional Supplementary Files [file 41467_2018_2877_MOESM3_ESM.pdf]

Description of Additional Supplementary Files

File Name: Supplementary Movie 1
Description: Step 1 of chlor-alkali processes using $Na_{0.44}MnO_2$ as redox mediator at an applied current of 100mA.

File Name: Supplementary Movie 2
Description: Step 2 of chlor-alkali processes using $Na_{0.44}MnO_2$ as redox mediator at an applied current of 100mA.

File Name: Supplementary Movie 3
Description: Step 1 of chlor-alkali processes using $Na_{0.44}MnO_2$ as redox mediator at an applied current of 1000mA.

File Name: Supplementary Movie 4
Description: Step 2 of chlor-alkali processes using $Na_{0.44}MnO_2$ as redox mediator at an applied current of 1000mA.

File Name: Supplementary Movie 5
Description: Step 1 of chlor-alkali processes using activated carbon as redox mediator at an applied current of 100mA.

File Name: Supplementary Movie 6
Description: Step 2 of chlor-alkali processes using activated carbon as redox mediator at an applied current of 100mA.
